# Recognizability bias in citizen science photographs

**DOI:** 10.1098/rsos.221063

**Published:** 2023-02-01

**Authors:** Wouter Koch, Laurens Hogeweg, Erlend B. Nilsen, Robert B. O’Hara, Anders G. Finstad

**Affiliations:** ^1^ Department of Natural History, Norwegian University of Science and Technology, 7491 Trondheim, Norway; ^2^ Department of Mathematical Sciences, Norwegian University of Science and Technology, 7491 Trondheim, Norway; ^3^ Norwegian Biodiversity Information Centre, Havnegata 9, 7010 Trondheim, Norway; ^4^ Intel Benelux, High Tech Campus 83, 5656 AE Eindhoven, The Netherlands; ^5^ Naturalis Biodiversity Center, PO Box 9517, 2300 RA Leiden, The Netherlands; ^6^ Norwegian Institute for Nature Research, Postboks 5685 Torgarden, 7485 Trondheim, Norway; ^7^ Faculty of Biosciences and Aquaculture, Nord University, Steinkjer, Norway

**Keywords:** citizen science, image recognition, machine learning, recognizability

## Abstract

Citizen science and automated collection methods increasingly depend on image recognition to provide the amounts of observational data research and management needs. Recognition models, meanwhile, also require large amounts of data from these sources, creating a feedback loop between the methods and tools. Species that are harder to recognize, both for humans and machine learning algorithms, are likely to be under-reported, and thus be less prevalent in the training data. As a result, the feedback loop may hamper training mostly for species that already pose the greatest challenge. In this study, we trained recognition models for various taxa, and found evidence for a *‘recognizability bias’*, where species that are more readily identified by humans and recognition models alike are more prevalent in the available image data. This pattern is present across multiple taxa, and does not appear to relate to differences in picture quality, biological traits or data collection metrics other than recognizability. This has implications for the expected performance of future models trained with more data, including such challenging species.

## 1. Introduction

There is an ever growing need for large amounts of biodiversity observation data. With an increasing awareness of the multiple crises biodiversity faces [1–3], substantial amounts of such data are essential if humanity is to monitor trends and address these issues [4–6]. Occurrence data are typically subject to spatial, temporal and taxonomic bias [7,8], and traditional manual methods of data collection are insufficient to gather the data volume needed, or address these biases. Alternative data collection methods, ranging from citizen science (non-professional volunteers reporting observations [9]) to camera-traps automating insect monitoring [10,11] are being deployed to gather large amounts of data. With the increased output from such initiatives, manual management and quality control become infeasible. Automated image recognition tools for species identification are increasingly used to facilitate this [12–15]. Image recognition models, however, bring with them a number of biases themselves that need to be addressed [16,17], and their training requires a large number of images [18]. This creates a mutual reliance between large-scale image data collection and image recognition models [19].

Visual identification of species is often a complex task, and taxa vary in their recognizability; while some species are unmistakable, many others are very challenging or even impossible to identify consistently, regardless of picture quality [20]. As models are trained using training data reported and identified by humans, species with low recognizability will often be under-reported and thus be under-represented in the training data. This affects recognition models, as these are then being trained with data biased towards taxa with higher recognizability. If this is the case, model performances will be hampered not only by the lower recognizability of particularly challenging species, but also by their lower presence in the training data.

To evaluate the existence of this possible bias and its consequences, we evaluated how data availability, picture quality, biological traits and data collection differs across species within three orders of birds, and how these differences relate to recognition model performance. All data came from a large Norwegian citizen science project, where recognition tools are not a part of the reporting or validation process. Birds are the most well-represented orders per species, allowing for the most detailed analysis. We also trained models for nine other orders of plants, animals and fungi, to test for a general correlation between data availability and model performance, and to evaluate what this means for future recognition models.

## 2. Methods

We trained image recognition models using convolutional neural networks on pictures retrieved from the Norwegian citizen science platform Species Observation Service [21] for 12 orders: Agaricales, Anseriformes, Asparagales, Asterales, Charadriiformes, Coleoptera, Diptera, Lecanorales, Lepidoptera, Odonata, Passeriformes and Polyporales [22]. This citizen science platform has no mobile interface, and relatively few pictures are made with mobile phones. For each of these orders, a separate model was trained using 200 observations with images per species for training and validation, and a minimum of 20 observations with images for the test set. This fixed amount of 220 observations per species was chosen to ensure a measurement of relative performances in models that contain no information about the actual numbers of observations in the dataset. Note that this is not a restriction one would impose on a model optimized for absolute performance. Earlier pilot runs executed on eight taxa (Bombus, Cetoniidae, Coccinellidae, Coleoptera, Lepidoptera, Odonata, Rodentia and Zygaenidae) with various numbers of images per species, executed using both an Inception-ResNet-v2 [23] as well as an EfficientNetB3 [24] architecture for comparison, indicated that model performance stabilized between 150 and 200 images per species at the latest. The availability of 220 observations was used as a criterion here to ensure representative performance scores while including a wide range of species.

Convolutional neural networks based on Inception-ResNet-v2 [23] were trained using the images from 180 observations per species as training data, plus 20 such observations as validation data. A minimum of another 20 observations with images of each species were designated as the test set and not included in the training stage. Colour channels of images were normalized, and images were scaled to 256×256 pixels, cropping them to become square if needed. The training data were augmented through shearing, zooming, rotating and mirroring images. A dense classification layer using softmax activation replaced the top layer of the Inception-ResNet-v2 model as a new top layer. For the loss function we used standard categorical cross entropy loss. When the learning rate had reached its minimum and accuracy no longer improved on the validation data, training was stopped and the best performing model was saved. After training, each model was tested on the images from the test sets of all species. From these results and various external datasets, several relevant metrics were collected (table 1).

**Table 1.** Metrics collected for species within all orders

metric	definition
data availability	The total number of citizen science observations from the Norwegian citizen science platform Species Observation Service [21] for a species, containing one or more pictures. This is a more meaningful measure than simply the total number of pictures, as multiple pictures within an observation are not independent from one another and therefore do not add as much information as unique observations.
*F*_1_-score	The performance obtained for a species in a recognition model, defined as the harmonic mean of the precision and recall. The *F*_1_-score is a commonly used metric for model evaluation, as it is less susceptible to data imbalance than model accuracy and precision [18].
species in Norway	The number of species within an order that are present in Norway, according to the Norwegian Species Nomenclature Database [25].

More detailed analyses were done on the included bird orders; waterfowl (Anseriformes), shorebirds (Charadriiformes) and passerines (Passeriformes), as bird orders have the highest proportion of species in Norway represented in the dataset, and ample standardized available data on a range of biological traits allowing for a deeper analysis. For these analyses, a number of additional metrics were collected for the included bird species (table 2). Urbanness, hand-wing index, body mass and habitat openness were included as they can be proxies of detectability and behaviour, and can affect the circumstances under which photos are taken in ways that are not measured by our picture quality metric, yet still influence recognizability in the resulting images. Documentation rate, picture density and observation rate are all related to observer behaviour and/or observer type, which can have an effect on the likelihood of the submission of an observation of a species with images, and the nature of these images.

**Table 2.** Metrics collected for species within the bird orders

metric	definition
picture quality	Using Label Studio v. 1.4 [26], greater than or equal to 50 pictures per species were annotated by drawing rectangles approximately equal in surface area to the visible part of each individual bird. From this, we took the percentage of the picture occupied by the largest depiction of an individual of the target species, minus the percentage of the picture occupied by all individuals of other bird species. As all pictures are scaled to equal dimensions this reflects the number of informative pixels for recognition regardless of original image size. Per species, the median log value was used as a proxy for picture quality.
urbanness	The proportion of 100 documented observations from the Species Observation Service with a location within a cell tagged as ‘urban’ in the ESA CCI landcover dataset [27].
hand-wing index	Wing length minus wing width, a measure positively correlated with flight efficiency and dispersal ability of a species. Retrieved from the Global-HWI dataset [28].
body mass	The average log-transformed body mass of a species, retrieved from the Global-HWI dataset [28].
habitat openness	A three-step scale of the openness of the habitat of a species, retrieved from the Global-HWI dataset [28].
documentation rate	The proportion, per species, of observations in the Species Observation Service that have one or more pictures.
picture density	The average number of images per observation from the Species Observation Service, from those with at least one picture.
observation rate	The number of observations in the Species Observation Service dataset per observation in the TOV-e bird monitoring scheme [29].

Least absolute shrinkage and selection operator (LASSO) multiple regression models were trained using Scikit-learn [30] to evaluate the effect of the biological traits, picture quality measurement and data collection process from table 2 on the *F*_1_-scores for birds. All LASSO models have the order as a factor. The full model for biological traits is given by

$$F_{1}=\beta_{0}+\beta_{1}\mathrm{HWI}+\beta_{2}\mathrm{BM}+\beta_{3}H+\beta_{4}U+\beta_{5}\mathrm{DA}+\epsilon +(1|\mathrm{Order}),$$

where HWI is the hand-wing index, BM is the body mass, *H* is the habitat openness, *U* is the urbanness and DA is the log data availability. The full model for picture quality is given by

$$F_{1}=\beta_{0}+\beta_{1}Q+\beta_{2}\mathrm{DA}+\epsilon +(1|\mathrm{Order}),$$

where *Q* is the picture quality and DA is the log data availability. The full model for data collection parameters is given by

$$F_{1}=\beta_{0}+\beta_{1}\mathrm{OR}+\beta_{2}\mathrm{DR}+\beta_{3}\mathrm{PD}+\beta_{4}\mathrm{DA}+\epsilon +(1|\mathrm{Order}),$$

where OR is the observation rate, DR is the documentation rate, PD is the picture density and DA is the log data availability.

## 3. Results

**Figure 1.**
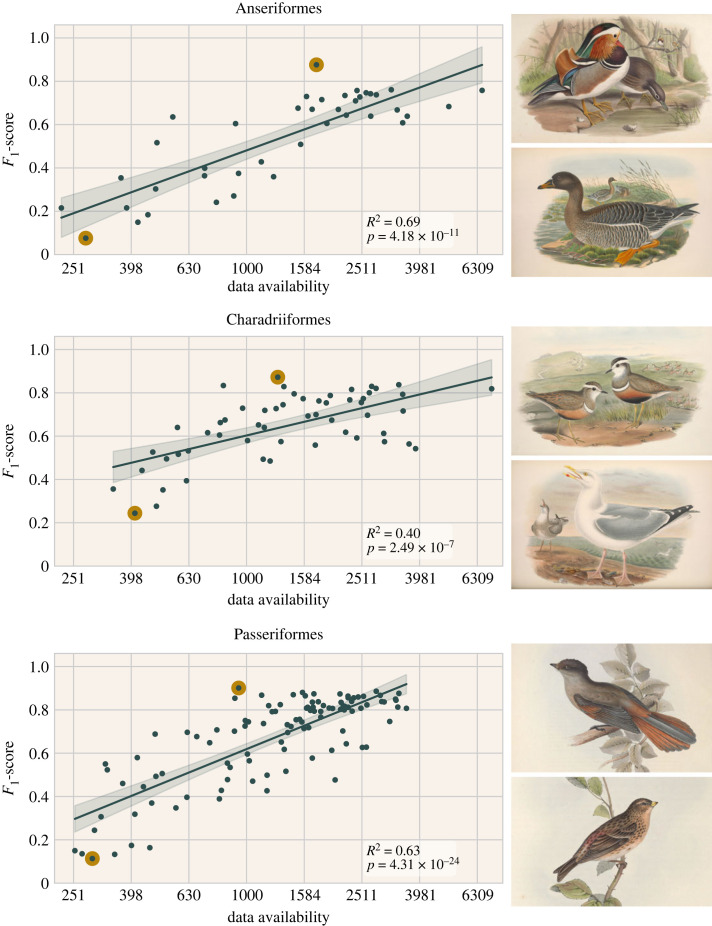
Effect of the total data availability per species on their *F*_1_-scores, in models trained with 200 documented observations, for three bird orders. The top- and bottom-performing species per order (highlighted dots) are depicted, see electronic supplementary material, table S1. Regressions are ordinary least squares with 95% CIs.

**Figure 2.**
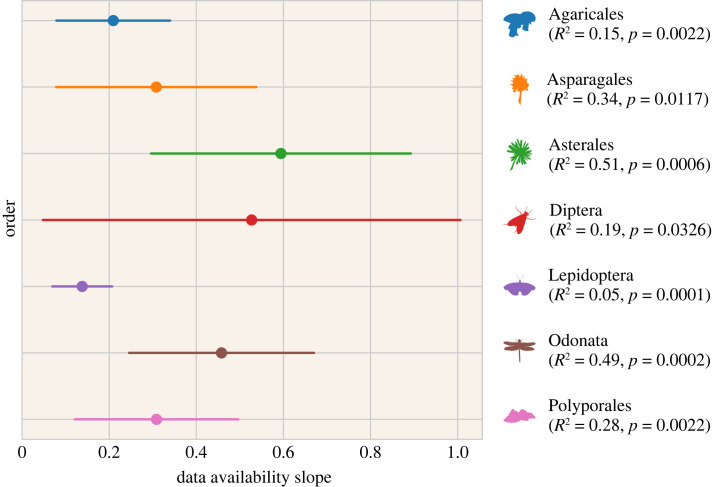
The slopes of the correlations between total data availability per species and their *F*_1_-scores, in models trained with 200 documented observations, for non-bird orders with a correlation *p* < 0.05. Regressions are ordinary least squares, lines indicate the 95% CIs.

Bird species on which the model performed better were also those that have the most observations with pictures available in our dataset; there was a strong positive linear correlation between log data availability and the *F*_1_-score for bird species (figure 1). Note that data availability did not affect training directly, as all models were trained and evaluated using 220 documented observations per species, regardless of the actual total availability. A positive linear correlation was also evident in seven of the nine non-avian orders (figure 2), in particular Asterales and Odonata. The beetles (Coleoptera) and lichens (Lecanorales) exhibited no apparent correlation, with an *R*^2^ of 0.06 and 0.12, and *p*-values of 0.27 and 0.18, respectively.

The *F*_1_-score increases observed are due to the increase in precision (*R*^2^ of 0.74, 0.56 and 0.70 for the Anseriformes, Charadriiformes and Passeriformes, respectively), not recall (*R*^2^ of 0.04, 0.08 and 0.02).

In each bird order, we found that species that were relatively more often documented with pictures also had more pictures per observation with images; there was a linear relationship between species’ picture density and documentation rate (*R*^2^ ≥ 0.52, *p* ≤ 1.51 × 10^−7^, see electronic supplementary material, table S2). We also found that species that had a higher average number of images per observation with images were more difficult for the recognition models; we observed a negative linear correlation between picture density and *F*_1_-scores (*R*^2^ ≥ 0.23, *p* ≤ 2.1 × 10^−4^, see electronic supplementary material, table S2). Species with relatively more observations with images also had lower recognition scores; there was some negative linear correlation between documentation rate and *F*_1_-scores (*R*^2^ ≥ 0.11, *p* ≤ 4.64 × 10^−3^, see electronic supplementary material, table S2). For passerines, pictures taken of species occurring in more open habitats were of lower quality; there was a negative linear relationship between habitat openness and picture quality (*R*^2^ = 0.26, *p* = 3.53 × 10^−8^, see electronic supplementary material, table S2). Waterfowl and shorebirds could not be evaluated in this regard, as they exclusively occur in open habitats.

None of the biological traits (urbanness, hand-wing index, body mass and habitat openness), collection process parameters (documentation rate, picture density and observation rate), nor picture quality improved predictions of recognition model performance; LASSO models trained on biological traits, collection process parameters and picture quality, all having log data availability as an additional parameter and order as a factor, had *R*^2^ values of 0.61, 0.57 and 0.63, respectively. Adding first-order interactions did not improve these LASSO models (giving *R*^2^ values of 0.62, 0.57 and 0.63, respectively). With that, none of the full LASSO model performances were substantial improvements from a LASSO model with log data availability as its only parameter (*R*^2^ = 0.57).

## 4. Discussion

We found a conspicuous pattern where recognition models attain higher performances for species that are reported with pictures more frequently. It is probable that the recognizability of the species influences both their likelihood of being reported with pictures, as well as recognition model performances. The citizen science project used as a data source here did not include any recognition tools in its reporting or validation process, allowing a distinction between human and algorithm recognition biases. Unmistakable species can be recognized and reported by more citizen scientists, resulting in greater image data availability for such species. A recognition model, dealing with the same information as human observers, is also proportionally more likely to reliably recognize these species. This is supported by the fact that the performance increase is due solely to the increased precision of the recognition models, as opposed to their recall. Models are less likely to erroneously suggest a distinct species, and will tend to suggest a less distinct species for images that are more difficult to classify.

Further support was found in a qualitative comparison between species with the highest and lowest recognition model performances, where easy to recognize, characteristic species were reported more often than hard to recognize species (e.g. nondescript species or species similar to other related species) (see figure 1 and electronic supplementary material, table S1). Furthermore, most of the correlation is explained by the data availability for a species, rather than the documentation rate or the picture density. Thus, there was more data available mainly when a species is recognized and reported more, rather than it being disproportionately more likely to be reported with pictures, or with many pictures when reported with pictures.

An alternative explanation to recognizability for increased model performance might be a difference in the kind of pictures, but we found no evidence for this. Species traits, habitat use and image quality could affect recognition model performance if pictures of more photographed birds are taken more up close, with higher zoom, or were cropped more. We found no evidence, however, for a link between model performance and either picture quality or biological traits in birds. For the passerines, where habitat openness varies among species, we did find that picture quality decreases for species associated with more open habitats. It makes intuitive sense that birds in open habitats are photographed from a greater distance than their forest dwelling counterparts, which will be hidden from view unless in close proximity. While this intercorrelation supports the validity of the picture quality metric, neither habitat nor picture quality affected recognition model performance. Based on these results we conclude that differences in model performance are caused by the recognizability of the species, rather than by how, or how large species are generally depicted.

Since multiple pictures connected to a single observation are not truly independent, training data are generated based on the number of documented observations, rather than the total number of pictures. One might expect that species with a higher picture density will perform better, as observations with more pictures can provide some additional information in the training process. We observed a reverse effect, however, where performance for such species was substantially lower. A likely explanation is that species with high picture densities are rarities in Norway (e.g. the top three species being Caspian gull, Blyth’s reed warbler and Pine bunting). Species with the lowest picture density, meanwhile, are typical common, well-known species such as corvids and titmice. Rarities are reported not because they are easy to find or identify by casual observers, but due to their popularity among avid birdwatchers, who are likely to document their observations. A strong correlation between picture density and documentation rate supports this; rarities are more often reported with pictures, and in such cases relatively often with several pictures.

While we investigated the bird orders in detail, the link between data availability and model performance was present also in non-avian orders (figure 2). Some orders are notoriously difficult to identify to species level, e.g. flies (Diptera) and beetles (Coleoptera), but our models for these groups performed surprisingly well. The list of species with sufficient observations with pictures for inclusion in the experiment reveals that only relatively easy to recognize species, often with distinct colorations (e.g. ladybugs for beetles) are represented in this subset.

Balancing the data to 220 citizen science observations with pictures per species is a limitation that one would not impose on a model aiming for best possible performance, but we know of no mechanism where this limitation would disproportionately affect certain species more than others. Furthermore, recognition models are usually subject to limited data availability, making the observed effect relevant even if enhanced by the limitation in the training data.

More generally, the requirement that species must have at least 220 citizen science observations with pictures generates a non-random subset of species, and it differs greatly per order how selective this criterion is. Bird species are most frequently reported; 48% of the species present in Norway [25] within the bird orders examined here meet the selection criterion. One of the other orders for which the pattern was found, the dragonflies and damselflies (Odonata), have only 52 species in Norway, of which 44% met the criteria for inclusion. This is in stark contrast to the beetles (1% inclusion), and lichens (2% inclusion), where no clear correlation was found. It is reasonable to assume that for these taxa, the experiment only considers the most recognizable species. If observations were thousandfold, more challenging species could be included, giving a broader range in performances and possibly a similar positive correlation between model performance and data availability.

The consequence of the recognizability bias found here is that as more data are collected, ultimately providing the numbers of pictures needed to train models also on less reported species, one has to be wary of extrapolating current performance of recognition models to these expanded models; data that are lacking now are in part lacking because such species are harder to recognize. When such data are added in the future, the performance increase will probably not be as great as in the past. Besides citizen science, even methods where all detected observations are reported, such as automated insect camera traps and trail cameras, can still be subject to recognizability bias. There too, species that are less readily identified will result in more unidentifiable pictures, providing relatively less training data.

Image recognition tools play an increasingly important role in maintaining the quality of the large amounts of biodiversity data science and management require. There are limits to what can be identified from a picture, however, and identification tools are needed that rely on more than just pixel information. Models that take into account season, location, sound, etc. can be especially beneficial for difficult species. Still, there is no substitute for the taxonomic knowledge of experts. Preserving this knowledge, and making it available in the form of identification keys is vital. These can be powerful tools to more reliably identify challenging species, in tandem with automatic identification.

## Data Availability

All code is available through Zenodo at https://doi.org/10.5281/zenodo.6734696 [31]. Bird illustrations in figure 1 are works in the Public Domain made by John Gould (1804–1881), obtained through the Biodiversity Heritage Library [32–34]. The data are provided in electronic supplementary material [35].
